# Site-specific fluorescent labeling to visualize membrane translocation of a myristoyl switch protein

**DOI:** 10.1038/srep32866

**Published:** 2016-09-08

**Authors:** Sung-Tae Yang, Sung In Lim, Volker Kiessling, Inchan Kwon, Lukas K. Tamm

**Affiliations:** 1 Center for Membrane and Cell Physiology, University of Virginia, Charlottesville, VA 22908, USA; 2 Department of Molecular Physiology and Biological Physics, University of Virginia School of Medicine, Charlottesville, VA 22908, USA; 3 Department of Chemical Engineering, University of Virginia, Charlottesville, VA 22904, USA; 4 School of Materials Science and Engineering, and Department of Biomedical Science and Engineering, Gwangju Institute of Science and Technology (GIST), Gwangju 61005, Republic of Korea

## Abstract

Fluorescence approaches have been widely used for elucidating the dynamics of protein-membrane interactions in cells and model systems. However, non-specific multi-site fluorescent labeling often results in a loss of native structure and function, and single cysteine labeling is not feasible when native cysteines are required to support a protein’s folding or catalytic activity. Here, we develop a method using genetic incorporation of non-natural amino acids and bio-orthogonal chemistry to site-specifically label with a single fluorescent small molecule or protein the myristoyl-switch protein recoverin, which is involved in rhodopsin-mediated signaling in mammalian visual sensory neurons. We demonstrate reversible Ca^2+^-responsive translocation of labeled recoverin to membranes and show that recoverin favors membranes with negative curvature and high lipid fluidity in complex heterogeneous membranes, which confers spatio-temporal control over down-stream signaling events. The site-specific orthogonal labeling technique is promising for structural, dynamical, and functional studies of many lipid-anchored membrane protein switches.

Fluorescence spectroscopy and microscopy are powerful methods to study interactions of biomolecules with membranes in cells and model systems. It is also possible to investigate dynamic interactions at the single molecule level by advanced fluorescence-based approaches^1^,^2^,^3^. However, fluorescent labeling of biomolecules still remains a challenge, because free cysteines, which are most frequently used for site-specific labeling, are often essential for a protein’s structure and/or function. Random fluorescent labeling at multiple lysines often also results in loss of the native structure and/or a protein’s function or its ability to reversibly bind to membranes. In order to overcome these issues, fluorescent labeling at only a single permissive site of a target protein is often required. A good example for an important non-constitutive membrane protein that exhibits such issues is the recoverin from the outer segments of rod and cone cells in the mammalian retina^4^. Recoverin is a Ca^2+^-sensor that reversibly binds to rhodopsin kinase depending on Ca^2+^ concentration and thereby inhibits phosphorylation and the lifetime of photoactivated rhodopsin^5^. An N-terminal myristoyl chain is sequestered in an interior pocket at low Ca^2+^, but is induced to protrude when Ca^2+^ binds to two binding sites on the protein. The myristoyl chain then acts as an anchor for the translocation of cytosolic recoverin to the rod outer segment disk membrane^6^,^7^, a process that is driven by hydrophobic interactions and further enhanced by lysine-mediated electrostatic interactions with the lipid bilayer^8^,^9^. Importantly, the free cysteine in position 39 (Cys39) is critically important for recoverin’s function and contributes to its ability to bind Ca^2+^. In fact, Cys39 is one of the most highly conserved residues and part of the CPXG motif in the Neuronal Calcium Sensor (NCS) family proteins^10^ and plays functional roles in redox sensing, dimerization, and ligand interactions^11^,^12^. For example, mutation of Cys39 to aspartic acid results in a significant reduction of photoreceptor membrane affinity^13^. Similarly, when Cys39 was labeled with the fluorophore Alexa647, the native membrane binding affinity of recoverin was compromised^14^.

Recoverin binds to membranes in a Ca^2+^-dependent manner as previously demonstrated by surface plasmon resonance spectroscopy and AFM-based force spectroscopy^15^,^16^. However, neither of these methods can be applied to measure translocation of the protein to membranes nor obtain dynamical information of this process at the single molecule level. Yet, many molecular details about the exact nature of recoverin-membrane interaction and its signaling dynamics remain to be elucidated. To address some of the unresolved issues, we describe here a site-specific labeling procedure that combines the genetic incorporation of a reactive non-natural amino acid with the application of bio-orthogonal chemistry. Importantly, this approach avoids touching the essential Cys39 and any of the 25 lysines, including at least 5 functionally important lysines near the N-terminus of recoverin that would be randomly modified by amino-reactive labeling techniques^17^. The approach also avoids targeting the N-terminus by reductive alkylation^18^ or native chemical ligation^19^,^20^, which are not suitable either because the N-terminus of recoverin is post-translationally modified by a functionally important myristoyl chain.

Our approach to achieve efficient heterologous expression of recoverin bearing both a functional *p*-azido-L-phenylalanine (AZF) group for small molecule fluorescent labeling or chemical ligation with a fluorescent protein and an N-myristoyl chain involves a genomically amber-free *E. coli* variant, in which release factor 1 was knocked out, as a robust expression host^21^. Upon labeling with the fluorescent dye 4-chloro-7-nitrobenzofurazan (NBD-chloride), DBCO-PEG_4_-carboxyrhodamine, or mCherry, we monitored and visualized Ca^2+^-dependent binding of recoverin to membranes and showed a strong effect of membrane curvature on its binding affinity. In addition, we demonstrated the spatial orientation of membrane-anchored recoverin in the lipid bilayer through dye labeling at distantly positioned sites and measured its partitioning between ordered and disordered lipid phases in heterogeneous membranes. Since many proteins have similar limitations for fluorescent labeling as recoverin, the strategy proposed here will likely be general and prove beneficial for examining a large group of protein-membrane interactions.

## Results

### Screening dye-labeling sites in recoverin

**Figure 1:**
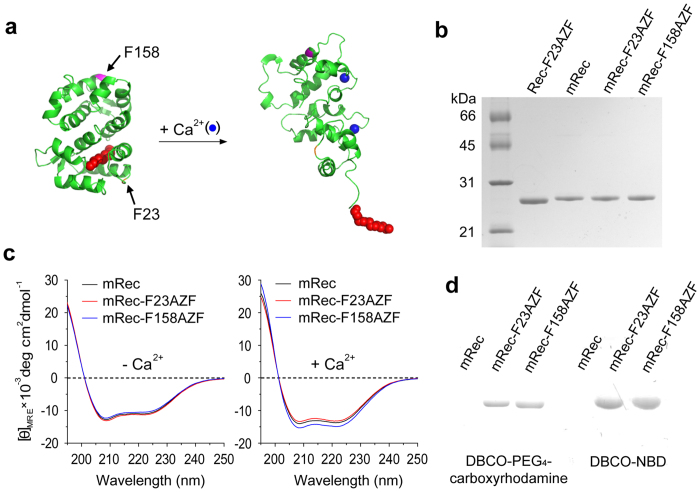
Genetic incorporation of the non-natural amino acid AZF into two selected positions of recoverin. (**a**) Three dimensional structures of recoverin in its Ca^2+^-free (PDB code: 1IKU; left) and Ca^2+^-bound (PDB code: 1JSA; right) forms. Sites selected for mutation are marked with arrows. The N-myristoyl chain is represented by red spheres. (**b**) SDS-PAGE of purified recoverin and variants: Lane 1, non-myristoylated Rec-F23AZF; Lane 2, myristoylated recoverin; Lane 3, mRec-F23AZF; Lane 4, mRec-F158AZF. (**c**) Far-UV CD spectra of recoverin and variants in the absence or presence of 1 mM Ca^2+^. (**d**) In-gel fluorescence of recoverin and variants reacted with DBCO-PEG_4_-carboxyrhodamine (left) or DBCO-NBD (right): Lane 1, mRec; Lane 2, mRec-F23AZF; Lane 3, mRec-F158AZF. The labeled protein bands run at 24 kDa as shown with the mCherry reference in Supplementary Fig. 4.

Recoverin undergoes a drastic conformational change upon binding of two Ca^2+^ ions at EF-hands 2 and 3, leading to the extrusion of an N-terminal myristoyl group and exposure of a hydrophobic groove^6^. To minimize perturbation of this important functional switch, potential sites for non-natural amino acid (NNAA) incorporation were examined in the context of physicochemical and functional constraints. The NNAA AZF is a phenylalanine analog with an azide substituted in *para* position. Since AZF has a similar chemical structure and hydrophobicity as phenylalanine and tyrosine, we considered all fourteen phenylalanines and five tyrosines of the native amino acid sequence of recoverin as potential candidates for AZF substitution. It has been reported that the efficiency of dye labeling largely depends on the solvent accessibility of targeted amino acid residues^22^. As assessed by the ASA-View Calculator^23^, each candidate exhibited varying degrees of the relative solvent accessibility depending on the position and the Ca^2+^-induced conformational change (Supplementary Fig. 1). Two phenylalanines in positions 23 (F23 in EF-1) and 158 (F158 in EF-4) were selected for mutation since they have both relatively high solvent accessibilities in the Ca^2+^-free and Ca^2+^-bound states, and neither of them is directly involved in the membrane-binding (Fig. 1a).

### Site-specific incorporation of *p*-azido-L-phenylalanine into myristoylated and non-myristoylated recoverin in genomically recoded *Escherichia coli*

Site-specific incorporation of NNAAs into target protein by cognate pairs of amber suppressor tRNAs and amino-acyl tRNA synthetases in bacterial expression systems inevitably suffers from low yield because the endogenous release factor 1 competitively binds to amber codons and terminates protein translation. Moreover, genomic amber codons are potential off-targets for NNAA-charged amber suppressor tRNAs, leading to a further reduction in expression yield and to deleterious effects on cell physiology. Recently, extensive genome engineering produced the genomically recoded *E. coli* strain C321.ΔA.exp, in which all amber codons have been replaced by ochre codons (UAA) thereby allowing for the release factor 1 to be deleted without impairing the native fitness of *E. coli*^21^. We exploited C321.ΔA.exp as host for the efficient site-specific incorporation of AZF into sites 23 and 158 of non-myristoylated recoverin by transforming it with expression vectors encoding recoverin with amber codons in the two respective positions and an orthogonal pair of amber suppressor tRNA/amino-acyl tRNA synthetase reactive towards AZF^24^. For incorporation of AZF into myristoylated recoverin, the host was transformed with an additional vector encoding N-myristoyl-transferase which co-translationally catalyzed the attachment of a myristic acid to the N-terminal glycine residue of recoverin^25^.

Three species containing AZF were expressed and purified to high purity: non-myristoylated recoverin with AZF in position 23 (Rec-F23AZF), myristoylated recoverin with AZF in position 23 (mRec-F23AZF), and myristoylated recoverin with AZF in position 158 (mRec-F158AZF) (Fig. 1b). To verify incorporation of AZF and N-myristoylation, proteins were subjected to MALDI-TOF mass spectrometry after tryptic digestion. Compared to wild-type, peptidic fragments containing AZF in positions 23 or 158 exhibited mass to charge ratios (*m/z*) that were increased by 15 units, which is less than the expected *m/z* shift of 41 upon substitution of AZF for phenylalanine (Supplementary Fig. 2). The discrepancy probably results from laser-induced degradation of the azide (–N_3_) into an amine (–NH_2_) group in the MALDI-TOF analyses^26^. As confirmed previously, the reduced masses do not result from metabolic conversion during fermentation^27^,^28^,^29^,^30^,^31^,^32^. To assess potential changes in secondary structure incurred by AZF incorporation we recorded far-UV circular dichroism (CD) spectra. No substantial differences were observed between wild-type and variants in the absence or presence of 1 mM Ca^2+^ (Fig. 1c). Derived contents of secondary structure are summarized in Supplementary Table 1. In order to validate the bio-orthogonal reactivity of a strained alkyne towards AZF, recoverin variants and wild-type were reacted with dibenzo-cyclooctyne (DBCO)-derivatized carboxy-rhodamine (Supplementary Fig. 3a). In contrast to wild-type, the reacted variants exhibited strong fluorescence in SDS polyacrylamide gels, demonstrating chemo-selective labeling and incorporation of carboxy-rhodamine into the proteins by bio-orthogonal chemistry (Fig. 1d, left).

### Synthesis and conjugation of DBCO-derivatized NBD

4-chloro-7-nitro-1,2,3-benzoxadiazole (NBD) is an environment-sensitive fluorescent probe that has been widely used to label biomolecules for investigating their interactions with artificial or cellular membranes^33^. To site-specifically attach NBD to recoverin through strain-promoted azide-alkyne cycloaddition (SPAAC)^24^,^34^, DBCO-functionalized NBD (DBCO-NBD) was synthesized by reacting NBD-chloride with DBCO-amine. We then used DBCO-NBD (Supplementary Fig. 3b) to label recoverin variants at both modified positions, yielding mRec-F23NBD, mRec-F158NBD, and Rec-F23NBD. Bio-orthogonality of NBD conjugation through SPAAC was verified by analyzing the fluorescence of the products in SDS gels (Fig. 1d, right). Residual dyes that might produce non-specific, false-positive signals in the following recoverin-membrane interaction study were removed by subjecting the reaction mixture to PD-10 column chromatography. We confirmed the absence of residual dyes in recoverin samples by in-gel fluorescence (Supplementary Fig. 4).

### Ca^2+^-dependent association of recoverin with membranes

**Figure 2:**
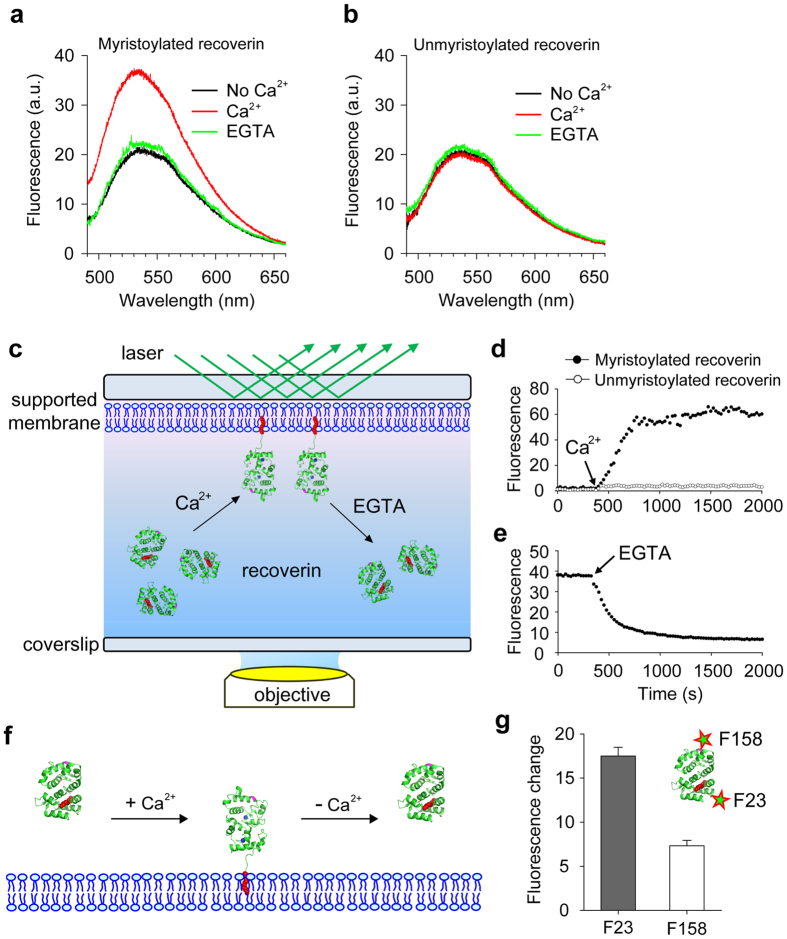
Ca^2+^-dependent binding of myristoylated recoverin to membranes. NBD-labeled myristoylated (mRec-F23NBD) and unmyristoylated (Rec-F23NBD) versions of recoverin were used for spectrometric measurements of membrane association. Fluorescence emission spectra of 0.1 μM mRec-F23NBD (**a**) and Rec-F23NBD (**b**) were recorded in LUVs (0.1 mM total lipids) composed of PC/PE (7:3 mol:mol) in the absence (black) and presence (red) of 1 mM Ca^2+^. 2 mM EGTA was added after 1 h incubation of recoverin with 1 mM Ca^2+^ (green). (**c**) Schematic of TIRF microscopy approach used to monitor the association and dissociation of recoverin to supported membranes. (**d**) Binding of 0.1 μM mRec-F23NBD and Rec-F23NBD to supported membranes composed of PC:PE (7:3 mol:mol). Mean fluorescence intensity over time, indicating recoverin binding to the membrane after addition of 1 mM Ca^2+^. (**e**) Dissociation of mRec-F23NBD from supported membranes. Mean fluorescence intensity over time, indicating recoverin dissociation from the membrane after addition of 2 mM EGTA. (**f**) Schematic diagram of recoverin-membrane interaction. The myristoyl group of recoverin is exposed by the Ca^2+^ binding and inserts into the membranes. Such recoverin binding can be dissociated from the membrane by Ca^2+^ removal. (**g**) Position-dependent NBD fluorescence intensity of membrane-bound recoverin. Fluorescence changes of mRec-F23NBD and mRec-F158NBD by Ca^2+^ addition were measured. Data are representative of three experiments in a, b, d, and e, and mean ± s.d. of triplicates in g.

To examine the role of the N-terminal myristoylation of recoverin in the Ca^2+^-dependent membrane association, we mixed unmyristoylated (Rec-F23NBD) and myristoylated (mRec-F23NBD) NBD-labeled recoverin with large unilamellar vesicles (LUVs) composed of phosphatidylcholine (PC) and phosphatidylethanolamine (PE) in the absence and presence of Ca^2+^. Myristoyl-Rec-F23NBD exhibited an increased fluorescence intensity and a blue shifted emission maximum in the presence of Ca^2+^ (Fig. 2a), indicating a strongly Ca^2+^-dependent binding of recoverin to these membranes. However, no significant change was observed for Rec-F23NBD upon addition of Ca^2+^ (Fig. 2b), suggesting that the myristoyl moiety of recoverin is responsible for membrane binding.

Next, we used total internal reflection fluorescence (TIRF) microscopy to monitor in real time the Ca^2+^-dependent binding of recoverin to planar supported membranes (Fig. 2c). Addition of Ca^2+^ resulted in a significant fluorescence increase by association of mRec-F23NBD to the membranes, whereas no fluorescence increase was detected with unmyristoylated Rec-F23NBD or free NBD dye (Fig. 2d and Supplementary Fig. 5). Membrane binding was reversible as bound recoverin was dissociated by the addition of EGTA (Fig. 2e). Therefore our site-specific labeling strategy with NBD demonstrates that the reversible binding of recoverin to membranes depends on Ca^2+^ and the N-terminal myristoyl anchor of recoverin, which is in agreement with previous findings^15^,^35^, but offers a much more sensitive probe that could also be used in fluorescence imaging in model membranes (Fig. 2f). To verify the predicted orientation of membrane-bound recoverin, we used myristoylated recoverin that was labeled with NBD in position 158 (mRec-F158NBD). This site is far from the site of myristoylation. The fluorescence intensity from this site was significantly lower than the intensity from NBD in position 23 (Fig. 2g and Supplementary Fig. 6), indicating that position 23 is most likely closer and in a generally more hydrophobic environment when binding to lipid bilayers than position 158. It is well known that the fluorescence of NBD strongly depends on the polarity of its environment and is much higher when inserted into or close to membrane surfaces^33^. This also supports the notion that recoverin binds to membranes with its myristoyl group inserted into the lipid bilayer.

### Curvature-dependent association of recoverin with membranes

**Figure 3:**
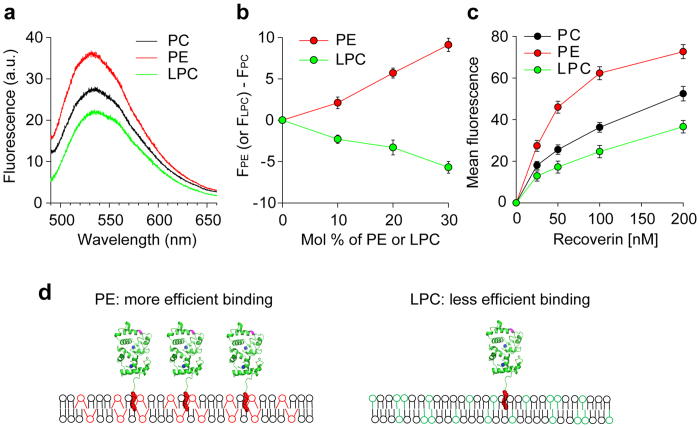
Effect of spontaneous membrane curvature on membrane association of recoverin. (**a**) Fluorescence emission spectra of 0.1 μM mRec-F23NBD were recorded in the presence of LUVs (0.1 mM total lipids) composed of PC, PC:PE (7:3), or PC:LPC (7:3) and in the presence of 1 mM Ca^2+^. (**b**) Relative fluorescence intensities measured as a function of the mol fractions of PE (red) or LPC (green) in PC bilayers at excitation and emission wavelengths of 475 and 530 nm, respectively. (**c**) Mean fluorescence intensity recorded by TIRF microscopy for the binding of mRec-F158NBD to supported membranes composed of PC, PC:PE (7:3), or PC:LPC (7:3) in the presence of 1 mM Ca^2+^. (**d**) Schematic diagram of lipid-dependent association of recoverin to membranes. Recoverin binds more efficiently to membranes containing cone-shaped lipids (PE) than inverted cone-shaped lipids (LPC). Data are representative of three experiments in a, and mean ± s.d. of triplicates in b and c.

Recoverin associates with retinal rod outer segment disc membranes, which contain high concentrations of PE lipids^36^. Since the presence of cone-shaped PE contributes to negative spontaneous curvature in membranes, we next examined the effect of PE and lipids introducing positive spontaneous curvature such as lysophosphatidylcholine (LPC) on the capacity to bind recoverin. When mRec-F23NBD was added to LUVs composed of only PC, PC:PE (7:3), or PC:LPC (7:3) in the presence of Ca^2+^, PC:PE exhibited an enhanced fluorescence intensity, whereas PC:LPC showed a decreased intensity compared to pure PC membranes (Fig. 3a). Upon addition of mRec-F23NBD, the fluorescence intensity increased or decreased proportionally to the added PE or LPC concentrations in the membranes, respectively (Fig. 3b). In addition, we demonstrated that there was more efficient binding to PE- than to LPC-containing membranes by measuring equilibrium binding of mRec-F158NBD to supported membranes by TIRF microscopy, the fluorescence quantum yield of which is less sensitive to the depth of recoverin in lipid bilayer than that of mRec-F23NBD (Fig. 3c). These results indicate that recoverin favors membranes with negative spontaneous curvature over those with positive spontaneous curvature. Membranes with negative spontaneous curvature exhibit more loosely packed headgroups on their surfaces, which may provide excellent sites for insertion of the myristoyl group of recoverin. Recoverin binding could also stabilize such membranes (Fig. 3d).

### Visualization of recoverin binding to supported lipid bilayers

**Figure 4:**
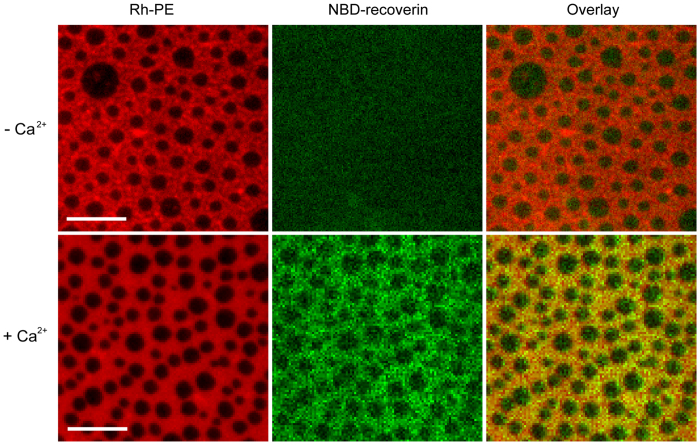
Visualization of Ca^2+^-dependent association of mRec-F23NBD to phase-separated supported membranes. Supported membranes with coexisting Lo/Ld phases (left panels) were composed of DPPC:DOPC:Cholesterol (2:2:1). The membranes were labeled with 0.1 mol% Rh-PE, which preferentially partitions into the Ld phase. Myristoyl-Rec-F23NBD was added to the membranes in the absence (top row) and presence (bottom row) of 1 mM Ca^2+^ (middle panels). The overlay (right panels) shows that mRec-F23NBD (green) associates with the Ld phase (red) on supported membranes in the presence of Ca^2+^. Scale bars are 10 μm. Representative images of three experiments.

We next visualized directly the binding of recoverin to supported lipid bilayers composed of dipalmitoyl-phosphatidylcholine (DPPC), dioleoyl-phosphatidylcholine (DOPC), and cholesterol, i.e. a commonly used model membrane system that produces coexisting liquid-ordered (Lo) and liquid-disordered (Ld) lipid phases such as those that characterize “lipid rafts” in cell membranes. It is recognized that the Lo phase is rich in saturated lipids and cholesterol which are highly ordered, whereas the Ld phase is rich in unsaturated lipids which are less well ordered^37^,^38^. The coexisting Lo/Ld phases on supported lipid bilayers were visualized by epifluorescence microscopy by doping the membrane with 0.1 mol% Rh-PE that preferentially partitions into Ld phases^39^. After 1 h incubation of the membrane with mRec-F23NBD in the absence and presence of Ca^2+^, membrane-bound recoverin was imaged by epifluorescence microscopy (Fig. 4). We only observed detectable NBD fluorescence signals in the presence of Ca^2+^, further supporting the Ca^2+^-dependent binding of recoverin to the membrane. The overlay image in Fig. 4 shows that recoverin associated more efficiently with Ld phase regions of the membrane than Lo phase regions, suggesting that the tight packing of saturated lipids and cholesterol in the Lo phase prevents the insertion of the myristoyl group into this lipid phase.

### Ca^2+^-responsive reversible translocation of recoverin site-specifically conjugated with mCherry

**Figure 5:**
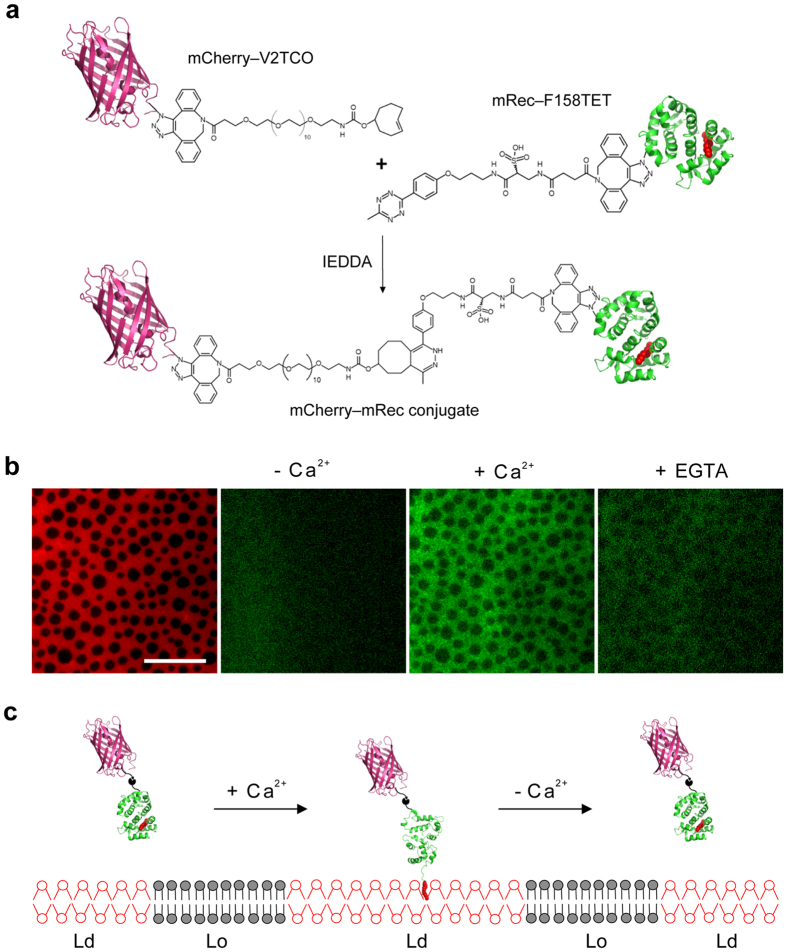
Synthesis of mCherry-recoverin conjugate and its Ca^2+^-responsive translocation to phase-separated supported membrane. (**a**) Schematic diagram of coupling between mCherry-V2TCO and mRec-F158TET. (**b**) Ca^2+^-dependent binding of the mCherry-recoverin conjugate to a supported membrane with ordered and disordered lipid domains. Supported membranes (left) were composed of DPPC:DOPC:Cholesterol (2:2:1) with coexisting Lo (dark) and Ld (red) phases. The membranes were labeled with 0.5 mol% DiD, which preferentially partitions into the Ld phase. The mCherry-recoverin conjugate was added to the membranes in the absence (center left) or in the presence (center right) of Ca^2+^. Addition of 2 mM EGTA (right) extracts most of the membrane-bound conjugate. Scale bar is 20 μm. Representative images of three experiments. (**c**) Schematic diagram depicting the reversible translocation of mCherry via the Ca^2+^-sensitive myristoyl chain (red) of recoverin into fluid phase regions of a phase-separated Lo/Ld membrane.

Spatiotemporal control of protein translocation to lipid membranes has great potential in the regulation of cellular behavior and the study of biological membrane structure and function. To develop a tool for such studies, we utilized recoverin as a molecular switch that delivers a target protein to the Ld phase of lipid membranes in a Ca^2+^-responsive manner. Site-specific incorporation of bio-orthogonally reactive NNAAs is useful for immobilizing proteins while retaining their native activity and controlling their spatial orientation^22^,^40^. We employed double-click chemistry^41^,^42^ to conjugate recoverin to mCherry as a model protein. To do so, we linked AZF that was genetically incorporated into mCherry or recoverin via SPAAC to a bifunctional linker consisting of a DBCO and a *trans*-cyclooctene (TCO) group with an intervening 12-subunit polyethylene (PEG_12_) linker, or a DBCO and a tetrazine (TET) group, respectively (Supplementary Fig. 3c,d). TCO and TET undergo rapid bio-orthogonal ligation by an inverse electron-demand Diels-Alder reaction (IEDDA). Since the IEDDA reaction is orthogonal to SPAAC as well, recoverin and mCherry can be conjugated via IEDDA subsequent to SPAAC in a fully chemo-selective and site-specific way. Valine 2 of mCherry was selected for NNAA modification with AZF to generate mCherry-V2AZF, which was then reacted with DBCO-PEG_12_-TCO to generate mCherry-V2TCO (Fig. 5a). Myristoyl-Rec-F158AZF was likewise reacted with DBCO-TET to generate mRec-F158TET. These two modified proteins were then conjugated via IEDDA (Fig. 5a). When run on an SDS gel, the reaction mixture showed an additional high molecular weight fluorescent band proving formation of a covalent linkage between mCherry and myristoylated recoverin (Supplementary Fig. 7).

The mCherry-recoverin conjugate was tested by TIRF microscopy for its ability to translocate in a Ca^2+^-responsive manner to supported membranes composed of DPPC:DOPC:cholesterol (2:2:1) (Supplementary Fig. 8a). Similar to recoverin alone, we observed that the conjugate associated with membranes upon addition of Ca^2+^ and dissociated from the membrane after subsequent addition of EGTA (Supplementary Fig. 8b,c). After washing away any unbound conjugates, the conjugate translocated to supported membranes with coexisting Lo/Ld phases was visualized by epifluorescence microcopy. No translocation of the conjugate was detected in the absence of Ca^2+^ while the addition of 1 mM Ca^2+^ triggered translocation of the conjugate to the Ld phase of phase-separated supported bilayers (Fig. 5b and Supplementary Fig. 9). After adding EGTA, much less fluorescence was observed, confirming that the conjugate translocated to the Ld phase regions in a reversible fashion. In addition, we measured the binding of the mCherry-recoverin conjugate, whose fluorescence intensity is insensitive to the depth of recoverin association with the lipid bilayer, to supported membranes containing the curvature promoting lipids PE and LPC (Supplementary Fig. 10). These experiments confirmed that recoverin interacts preferentially with PE-containing rather than LPC-containing membranes. The reversible Ca^2+^-dependent translocation to the more fluid and PE-rich regions of the membrane strongly suggest that the conjugate retained its functionality as an active myristoyl switch, capable of regulating the translocation of mCherry as a cargo protein to membranes in a spatio-temporal manner (Fig. 5c).

## Discussion

In this study, we developed a site-specific fluorescent labeling technique that minimizes conformational and functional changes of proteins featuring functionally important cysteines and lysines that are not available for modification with spectroscopic probes. Previously, non-natural amino acids including AZF have been genetically incorporated into membrane-bound proteins including receptors, transporters and ion channels, allowing for detailed investigations of ligand binding sites and other properties^43^,^44^,^45^,^46^,^47^. However, to our knowledge Ca^2+^-responsive switch proteins that conditionally interact with membranes have not so far been studied using non-natural amino acid incorporation and bio-orthogonal labeling techniques. Recoverin from rod outer segment disc membranes from mammalian retinal cells has an allosterically important cysteine and multiple lysines near its myristoylated N-terminus that are required for its Ca^2+^-dependent translocation to the disc membranes. Therefore, its functional translocation to membranes cannot be monitored by traditional labeling approaches and subsequent fluorescence spectroscopy or microscopy. However, the described approach of site-specifically incorporating the non-natural amino acid AZF into carefully selected sites and applying bio-orthogonal dye labeling strategies allowed us to measure the functional and Ca^2+^-dependent translocation of recoverin to membranes. Our results showed that this translocation depends on the curvature of the target membrane and the lipid packing density in that membrane. They also showed that recoverin preferentially translocates to liquid-disordered regions of model membranes with coexisting Lo- and Ld-phase domains. Finally, by using labeling sites in membrane-proximal and -distal positions, we were able to determine the molecular orientation of membrane-bound recoverin relative to the lipid bilayer.

The controlled localization of proteins to membranes is of great interest in cell biology and membrane biophysics^48^,^49^. Current techniques to engineer proteins for translocation to membranes include the introduction of hydrophobic transmembrane segments or fusing the protein of interest to membrane-binding domains by genetic manipulation or chemical ligation^50^,^51^,^52^. However, these approaches often lead to insoluble products and uncontrolled protein aggregation, and therefore, are frequently not amenable to a finely tuned spatio-temporal control of translocation^50^,^53^. Our strategy solves this problem by engineering specific labeling sites into virtually any position of the protein of interest without touching critical electrostatic regions or functionally important cysteines.

By applying the bio-orthogonal labeling strategy using genetic incorporation of non-natural amino acids, we produced several Ca^2+^-responsive fluorescent versions of recoverin that retained their ability to translocate to lipid bilayer membranes. The binding to membranes was fully reversible and the generated proteins did not suffer any solubility problems. The site-specific conjugation of recoverin to mCherry also demonstrates that our method could be used to attach a range of different cargo proteins to the myristoyl switch provided by recoverin. Therefore, our design offers a versatile platform to deliver multiple proteins of interest to membranes in a Ca^2+^-dependent fashion and thus provides flexible control for membrane translocation of such cargo proteins. The switchable delivery of proteins of interest to lipid membranes enabled by our approach should be very useful for studying a whole range of dynamic interactions of proteins with membranes. When combined with membranes that are spatially heterogeneous it is possible to deliver proteins of interest to the desired membrane compartments as demonstrated here for the targeting of recoverin to Ld phase regions of phase-separated membranes with co-existing Lo and Ld domains. Finally, by varying the site of non-natural amino acid incorporation and its fluorescent labeling as demonstrated here for two sites on recoverin, it is possible to control the molecular orientation of conjugated proteins relative to the lipid bilayer and investigate downstream effects of membrane orientation on cell signaling and the interaction of the test protein with other membrane components.

The functions of multiple regulatory pathways in neurons are tightly controlled by Ca^2+^ fluxes with strict spatial and temporal diversity and neuronal calcium sensor proteins like recoverin contribute a great deal to this exquisite Ca^2+^ control^54^. Recoverin and other NCS proteins are similar to each other and share globular shapes, N-terminal lipidation, and four EF-hand motifs, which harbor a critical cysteine and confer distinct Ca^2+^ binding sites to these proteins^55^. The myristic acid that is covalently attached to the N-terminal glycine allosterically responds to Ca^2+^-binding and thus is critically important for the association of NCS proteins with membranes. Combined with several basic residues near the N-terminus the myristoyl chain provides a Ca^2+^-dependent myristoyl switch to recoverin that is critically important for its function and, therefore, must not be disrupted by any spectroscopy labeling technique. Because of these many similarities, the bio-orthogonal labeling strategy developed here for recoverin should also prove useful for studying myristoyl-switching on membranes of many other NCS and presumably other proteins with complex signaling switches.

In summary, the site-specific bio-orthogonal labeling procedure described here provides a versatile platform for probing membrane interactions of many proteins that require spatio-temporal switchable control. The procedure is very general and should be helpful to answer many important questions of cell signaling in model and cell membranes that involve tightly controlled protein interactions on membranes.

## Methods

### Materials

*p*-azido-L-phenylalanine (AZF) was obtained from Chem-Impex International (Wood Dale, IL) and dissolved in 0.2 M NaOH to make 100 mM stock solution. Ni-NTA agarose and pQE80 plasmid were obtained from Qiagen (Valencia, CA). Amicon ultra centrifugal filters with a molecular weight cutoff of 10 kDa and ZipTip with C_18_ media were purchased from Millipore Corporation (Billerica, MA). Sequencing grade modified trypsin was obtained from Promega Corporation (Madison, WI). DBCO-PEG_4_-carboxyrhodamine, DBCO-amine, DBCO-PEG_12_-TCO, and DBCO-TET were purchased from Bioconjugate Technology Company (Scottsdale, AZ). Sodium myristate was obtained from TCI (Portland, OR) and dissolved in water at 55 °C to make 10 mM stock solution. Superdex 200 10/300 GL size exclusion column and PD-10 desalting columns were obtained from GE Health care (Piscataway, NJ). Biologic DuoFlow chromatography system and Mini-PROTEAN system for SDS-PAGE were obtained from Bio-Rad (Hercules, CA). A handcast gel with 1 mm thickness was made using the handcasting set included in Mini-PROTEAN system according to a standard protocol for a 12% polyacrylamide gel. The Biospectrum imaging system used to analyze in-gel fluorescence of fluorescently labeled proteins was obtained from UVP (Upland, CA). All lipids were from Avanti Polar Lipids (Alabaster, AL). 1,1′-dioctadecyl-3,3,3′,3′-tetramethylindodicarbocyanine perchlorate (DiD), were from Molecular Probes (Invitrogen, Carlsbad, CA). 1,2-dimyristoyl-phosphatidylethanolamine-N-(polyethylene glycol-triethoxysilane (DPS) were custom synthesized by Shearwater Polymers (Huntsville, AL). All chemicals were obtained from Sigma-Aldrich Corporation (St. Louis, MO) unless otherwise stated.

### Plasmid construction and bacterial strains

A plasmid pEVOL-AZF^56^ encoding an AZF-specific mutant pair of amber suppressor tRNA and tRNA synthetase derived from *Methanococcus jannaschii* was a gift from Peter Schultz (Addgene plasmid # 31186) (Cambridge, MA), and used without modification^24^. An expression plasmid encoding T7-promoted yeast N-myristoyltransferase (NMT), pNMT, was a gift from James Hurley (Addgene plasmid # 42578)^25^, and modified as described below. To replace the T7 promoter/terminator in pNMT with those compatible with endogenous *E. coli* RNA polymerase, the coding sequence of NMT was inserted into pQE80 in frame with both T5 promoter and t_0_ terminator by using primers NMTtoQE80 F and R (Supplementary Table 2). The entire region flanking T5-NMT-t_0_ was cloned back into pNMT by primers, 80toRSF F and R, substituting the original operon, and yielded pNMT-T5. N-myristoyltransferase, when coexpressed with recoverin, covalently adds a myristic acid to Gly at the N-terminus of recoverin. To construct an expression vector for recombinant recoverin and variants, its coding sequence was amplified from pTrec2^6^, a kind gift from James Ames (University of California, Davis, CA), by RecToQE80GFP F and R primers and cloned into pQE80 to give pQE80-Rec. Site-directed mutagenic PCR was performed with pQE80-Rec to replace the phenylalanine codon at amino acid positions 23 or 158 with an amber codon, yielding pQE80-Rec-F23amb and pQE80-Rec-F158amb. Two pairs of primers used for the mutagenic PCR were Rec F23amb F/R and RecF158amb F/R, respectively. To construct an expression vector for mCherry, its coding sequence was cloned into pQE80 with a hexa-histidine sequence between methionine-1 and valine-2 to give pQE80-mCherry using a pair of primers, Fusion RF F and R. Additionally, the codon for valine-2 was mutated to an amber codon for genetic incorporation of AZF to yield pQE80-mCherry-V2amb using a pair of primers, mCherryV2amb F and R. All DNA cloning in this study was performed by the restriction-free cloning technique^57^. As an expression host for the wild-type myristoylated recoverin (mRec), *E. coli* TOP10 was co-transformed with pQE80-Rec and pNMT-T5 to generate TOP10[mRec]. Genomically recoded *E. coli* C321.ΔA.exp was a gift from George Church (Addgene plasmid # 49018)^21^, and transformed with three plasmids, pEVOL-AZF, pNMT-T5, and pQE80-Rec-F23amb or pQE80-Rec-F158amb, generating C321.ΔA.exp[mRec-F23AZF] and C321.ΔA.exp[mRec-F158AZF], to produce myristoylated recoverin with a site-specifically incorporated AZF in positions 23 (mRec-F23AZF) or 158 (mRec-F158AZF). Non-myristoylated recoverin containing AZF in position 23 (Rec-F23AZF) was expressed from the same host without pNMT-T5. pQE, pNMT, pEVOL have ampicillin, kanamycin, and chloramphenicol resistance, respectively. Protein expression was induced by IPTG for pQE and pNMT, and by L-(+)-arabinose for pEVOL. All cloning was performed by the restriction free cloning technique^57^. The sequences of all primers used in this study are listed in Supplementary Table 2.

### Expression and purification of recoverin, mCherry, and their variants

To produce mRec, TOP10[mRec] was grown to saturation at 37 °C in 2×YT medium containing 100 μg/mL ampicillin and 50 μg/mL kanamycin with vigorous shaking at 220 rpm and then diluted 100 times into fresh medium. When an OD_600_ of 0.6 was reached, IPTG was added to a final concentration of 1 mM to induce protein expression and N-myristoylation. Cells were harvested after 5 hrs, and pelleted by centrifugation at 5,000 rpm for 10 min. Site-specific incorporation of AZF to produce mRec-F23AZF and mRec-F158 AZF was performed by inoculating a saturated culture of C321.ΔA.exp[mRec-F23AZF] or C321.ΔA.exp[mRec-F158AZF] into fresh 2×YT medium containing 0.1 mM sodium myristate as well as 100 μg/mL ampicillin, 50 μg/mL kanamycin, 35 μg/mL chloramphenicol at 37 °C. When an OD_600_ of 0.5 was reached, an AZF solution was added to a final concentration of 1 mM. After 10 min incubation, protein expression, AZF incorporation, and N-myristoylation were induced by 1 mM IPTG and 0.2% (w/v) L-(+)-arabinose. Cells were harvested after 8 hrs and pelleted by centrifugation at 5,000 rpm for 10 min. To extract and purify the proteins, the cell pellets were resuspended with lysis buffer consisting of 50 mM sodium phosphate (pH 7.5), 0.3 M NaCl, 10 mM imidazole, 1 mg/mL lysozyme, DNase, RNase, and a protease inhibitor cocktail, and mixed by rotation at 37 °C for 1 hr followed by at 4  °C for 2 hrs. After centrifugation at 11,000 rpm for 30 min, the clear supernatant was recovered, mixed with Ni-NTA agarose for 1 hr, and then washed with washing buffer consisting of 50 mM sodium phosphate (pH 7.5), 0.3 M NaCl, and 20 mM imidazole on a gravity-flow column to remove impurities. Proteins were eluted with elution buffer consisting of 50 mM sodium phosphate (pH 7.5), 0.3 M NaCl, and 250 mM imidazole, and then buffer-exchanged to PBS on a PD-10 column. Site-specific incorporation of AZF into mCherry was carried out as described above using *E. coli* C321.ΔA.exp harboring pQE-mCherry-V2amb and pEVOL-AZF.

### MALDI-TOF mass spectrometry

Proteins in PBS at 0.5 mg/mL were digested with trypsin in a final protease:protein ratio of 1:50 (w/w) by incubation at 37 °C overnight, and then desalted on a ZipTip C_18_ according to the manufacturer’s protocol. Purified tryptic digests mixed with DHB matrix (20 mg/mL of 2,5-dihydroxybenzoic acid and 2 mg/mL of L-(−)-fucose dissolved in 10% ethanol) at 1:1 (v/v) were subjected to mass characterization by Microflex (Bruker Corporation, Billerica, MA) MALDI-TOF mass spectrometry.

### Fluorescent dye labeling

DBCO-amine was reacted with 2 equivalents of NBD-chloride in DMSO at RT. After 1 hr, ethanol-amine was added to a final concentration of 1 M to quench residual NBD-chloride. Recoverin variants and DBCO-PEG_4_-carboxyrhodamine or DBCO-NBD were mixed in PBS at 1:4 molar ratios to a final concentration of 30 μM and 120 μM, respectively, and reacted at RT for 4 hrs. The mixture was directly loaded onto SDS-polyacrylamide gel for in-gel fluorescence analysis or desalted/buffer-exchanged on a PD-10 column. In order to remove residual dyes from labeled proteins, early-eluting fractions were collected for use in membrane binding experiment. For in-gel fluorescence analysis, the gel was illuminated at λ_ex_ = 480 nm, and the emitted light above 510 nm was collected through a SYBR Green emission filter. The exposure time was 2 s in all experiments. If necessary, labeled proteins were concentrated by passing over a centrifugal filter with a molecular weight cut-off of 10 kDa.

### Circular dichroism (CD) and data analysis

The secondary structures of mRec and its variants in 10 mM sodium phosphate (pH 7.0) were evaluated using a Jasco 710 spectropolarimeter (using a 1 mm path-length quartz cuvette) at room temperature at a protein concentration of 0.1 mg/mL. The background-subtracted sample spectra were then deconvolved to obtain numerical estimations of secondary structure contents using the DichroWeb online CD analysis server^58^, employing the SELCON3 analysis program along with the ‘Set #4′ reference set^59^.

### Site-specific conjugation of mCherry to recoverin

mCherry-V2AZF and mRec-F158AZF at 50 μM were individually reacted in PBS (5% DMSO v/v) with a 3-fold molar excess of DBCO-PEG_12_-TCO and DBCO-TET, respectively, at RT for 12 hrs and then desalted using a PD-10 column to remove residual linkers to obtain mCherry-V2TCO and mRec-F158TET. mCherry-V2TCO and mRec-F158TET were mixed at an equal molar ratio and then concentrated to a total protein concentration of 5 mg/mL using a centrifugal filter with molecular weight cut-off of 10 kDa. After 2 hrs at RT, the reaction mixture was analyzed by SDS-PAGE to confirm generation of the mCherry-recoverin conjugate. Size exclusion chromatography was performed on a Biologic DuoFlow chromatography system to isolate the conjugate from the reaction mixture.

### Binding of recoverin to lipid vesicles

Large unilamellar vesicles (LUVs) were prepared by extrusion through polycarbonate filters. In brief, the desired amount of lipids dissolved in chloroform or chloroform/methanol was evaporated under a stream of nitrogen gas in a glass test tube and further dried under high vacuum overnight. The lipid film was hydrated with HEPES buffer (10 mM HEPES, 150 mM NaCl, pH 7.2) and then vortexed. The resulting suspension was subjected to ten cycles of freezing and thawing and thereafter extruded 21 times through two stacked polycarbonate filters with pores of 100 nm in diameter (Avestin, Ottawa, ON). Binding of NBD-labeled recoverin to LUVs was measured as described^33^. In short, LUVs were added successively to 0.1 μM NBD-recoverin in HEPES buffer. The fluorescence emission spectra in vesicles of different lipid composition were recorded using a Fluorolog-3 spectrofluorometer (Jobin-Yvon, Edison, NJ) with the excitation and emission wavelengths set at 475 nm and 530 nm, respectively.

### Binding of fluorescenct proteins to planar supported membranes by TIRF microscopy

The fluorescence of NBD-labeled recoverin and mCherry-recoverin conjugate to the surface of a supported membranes was monitored on a Zeiss Axiovert 35 fluorescence microscope (Carl Zeiss), equipped with a ×40 water immersion objective (Carl Zeiss; numeric aperture = 0.7) and prism-based TIRF illumination. The prism-quartz interface was lubricated with glycerol to allow easy translocation of the sample cell on the microscope stage. The beam was totally internally reflected at an angle of 72° from the surface normal, producing an evanescent wave that decayed exponentially in the solution with a characteristic penetration depth of ∼100 nm. The light source was an argon ion laser (Innova 300C; Coherent, Palo Alto, CA) tuned to 488 nm for NBD or to 514 nm for mCherry. Fluorescence was observed through a 535 nm band pass filter (D535/40, Chroma, Brattleboro, VT) for NBD and 610 nm band pass filter (D610/60, Chroma, Brattleboro, VT) for mCherry by electron-multiplying CCDs (DU-860E or DV887ESC-BV, Andor Technologies). Mean fluorescence intensities of all camera pixels was recorded every 30 s to measure the association or dissociation of recoverin to/from supported membranes after the addition of 1 mM Ca^2+^ or 2 mM EGTA, respectively. To examine the effect of membrane curvature on association of recoverin with lipid bilayer membranes, the mean fluorescence intensity was measured after a 30 min incubation of mRec-F158NBD with supported membranes composed of PC:PE (7:3), or PC:LPC (7:3) in the presence of 1 mM Ca^2+^.

### Visualization of recoverin binding to phase-separated planar supported lipid bilayers

Supported lipid bilayers were formed by the combined Langmuir-Blodgett (LB)/LUV fusion technique as described previously^60^. In short, lipid mixtures composed of DPPC:DOPC:Cholesterol (2:2:1) with 3 mol% of DMPE-PEG-triethoxysilane were spread onto a pure water surface in a Nima 611 Langmuir-Blogdett trough (KSV NIMA, Espoo, Finland) and the surface pressure was compressed to 32.2 mN/m at a rate of 10 cm^2^/min. Quartz slides (Quartz Scientific, Fairport Harbor, OH) cleaned by Pirana solution (3:1 mixture of concentrated H_2_SO_4_ and H_2_O_2_) were dipped into the trough at a speed of 200 mm/min and were raised at a rate of 5 mm/min. The slide with transferred LB monolayer was placed in a custom-built flow-through chamber and 0.1 mM LUVs composed of DPPC:DOPC:Cholesterol (2:2:1) labeled with NBD-DPPE, Rh-PE, or DiD in HEPES buffer were injected into the chamber. After 2 hrs of incubation, excess LUVs were washed out by extensive rinsing with HEPES buffer. Phase assignments were made based on the previously established partitioning of several fluorescent lipid analogues^61^,^62^. Experiments were performed under conditions that limit potential effects of photodamage/oxidation on Lo phase formation as also previously established in those studies. To visualize the binding of recoverin to the supported membrane, 0.1 μM NBD-recoverin was incubated with the membrane in the absence or presence of Ca^2+^ at room temperature for 1 hr. Images were recorded on a Zeiss Axiovert 200 fluorescence microscope (Carl Zeiss, Thornwood, NY) equipped with a mercury lamp, a 40× water immersion objective (Carl Zeiss; NA = 0.7), and an electron multiplying charge-coupled device (EMCCD) cooled to −70 °C (iXon DV887ESC-BV, Andor, Belfast, U.K.) as a detector. NBD-labeled recoverin was illuminated through a 480 nm band-pass filter (D480/30, Chroma, Brattleboro, VT) and via a dichroic mirror (505dclp, Chroma) through the objective. Fluorescence was observed through a 535 nm band-pass filter (D535/40, Chroma). Rhodamine-stained bilayers and mCherry-recoverin conjugates were illuminated through a 540 nm band-pass filter (D540/25, Chroma) and via a dichroic mirror (565dclp, Chroma) through the objective. Fluorescence was observed through a 605 nm band-pass filter (D605/55, Chroma). DiD-stained bilayers were illuminated through a 620 nm (ET620/60, Chroma, Brattleboro, VT) and observed through a 665 nm band-pass filter (HQ665/60, Chroma, Brattleboro, VT). All images were obtained at room temperature.

## Additional Information

**How to cite this article**: Yang, S.-T. *et al.* Site-specific fluorescent labeling to visualize membrane translocation of a myristoyl switch protein. *Sci. Rep.* **6**, 32866; doi: 10.1038/srep32866 (2016).

## Supplementary Material

Supplementary Information
