# Supplementary material for: Site-specific fluorescent labeling to visualize membrane translocation of a myristoyl switch protein
Source: Sci Rep. 2016 Sep 8;6:32866. doi: 10.1038/srep32866 (PMC5015116; doi:10.1038/srep32866)
Supplement: Supplementary Information [file srep32866-s1.pdf]

Supplementary Information

Site-specific fluorescent labeling to visualize membrane translocation of a myristoyl switch protein

Sung-Tae Yang[1,2,5], Sung In Lim[3,5] , Volker Kiessling[1,2], Inchan Kwon[3,4,*], and Lukas K. Tamm[1,2,*]

[1]Center for Membrane and Cell Physiology, University of Virginia, Charlottesville, VA 22908, USA
[2]Department of Molecular Physiology and Biological Physics, University of Virginia School of Medicine, Charlottesville, VA 22908, USA
[3]Department of Chemical Engineering, University of Virginia, Charlottesville, VA 22904, USA
[4]School of Material Science and Engineering, and Department of Biomedical Science and Engineering, Gwangju Institute of Science and Technology (GIST), Gwangju 61005, Republic of Korea

[5]These authors contributed equally to this work.

***Corresponding authors:** Inchan Kwon (email: inchan@gist.ac.kr), or Lukas K. Tamm (email: Lkt2e@virginia.edu)

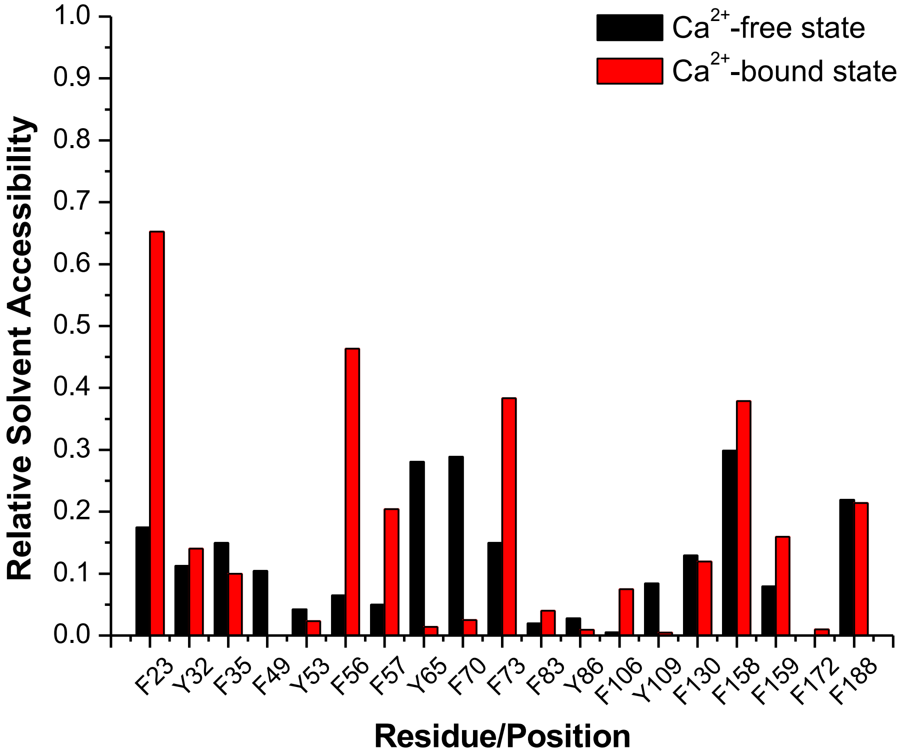

Supplementary Figure 1

Relative solvent accessibilities of potential sites for AZF incorporation into recoverin.

ASA-View Calculator, an online server for a graphical representation of residue-based solvent accessibility, was used to calculate the relative solvent accessibilities of all Phe and Tyr residues of recoverin based on their 3-dimensional structures (PDB accession codes: 1IKU for Ca^{2+}-free and 1JSA for Ca^{2+}-bound recoverin).

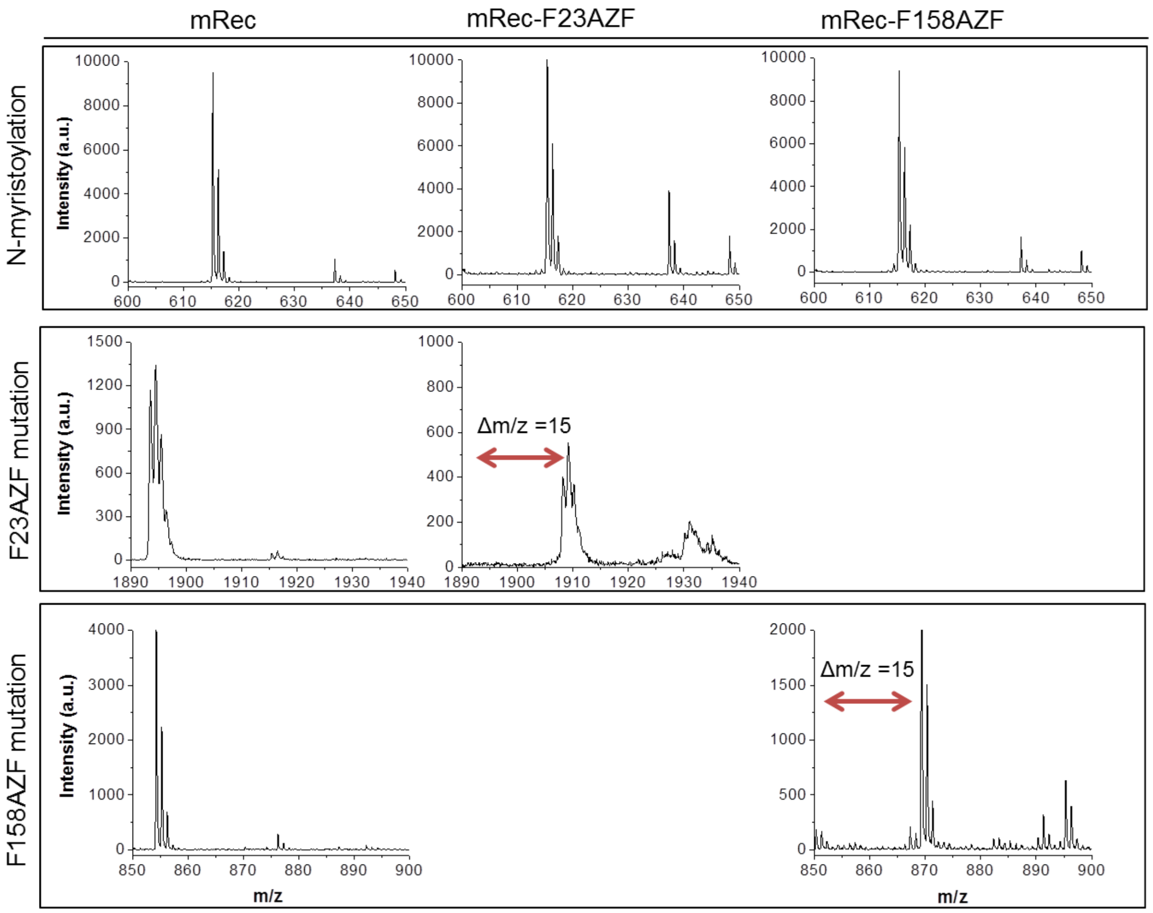

Supplementary Figure 2

MALDI-TOF mass spectra of tryptic digests of mRec and variants.

(Top) The GNSK peptide fragment corresponding to residues 2-5 has an expected *m/z* = 405 and exhibits an *m/z* of 615 (Δ*m/z* = 210), representing N-myristoylation. (Middle) The FTEEELSSWYQSFLK peptide fragment corresponding to residues 23-37 has an expected *m/z* = 1894 and exhibits an increased *m/z* due to the F23-to-AZF substitution. (Bottom) The IWGFFGK peptide fragment corresponding to residues 155-161 has an expected *m/z* = 854 and exhibits an increased *m/z* due to the F158-to-AZF substitution.

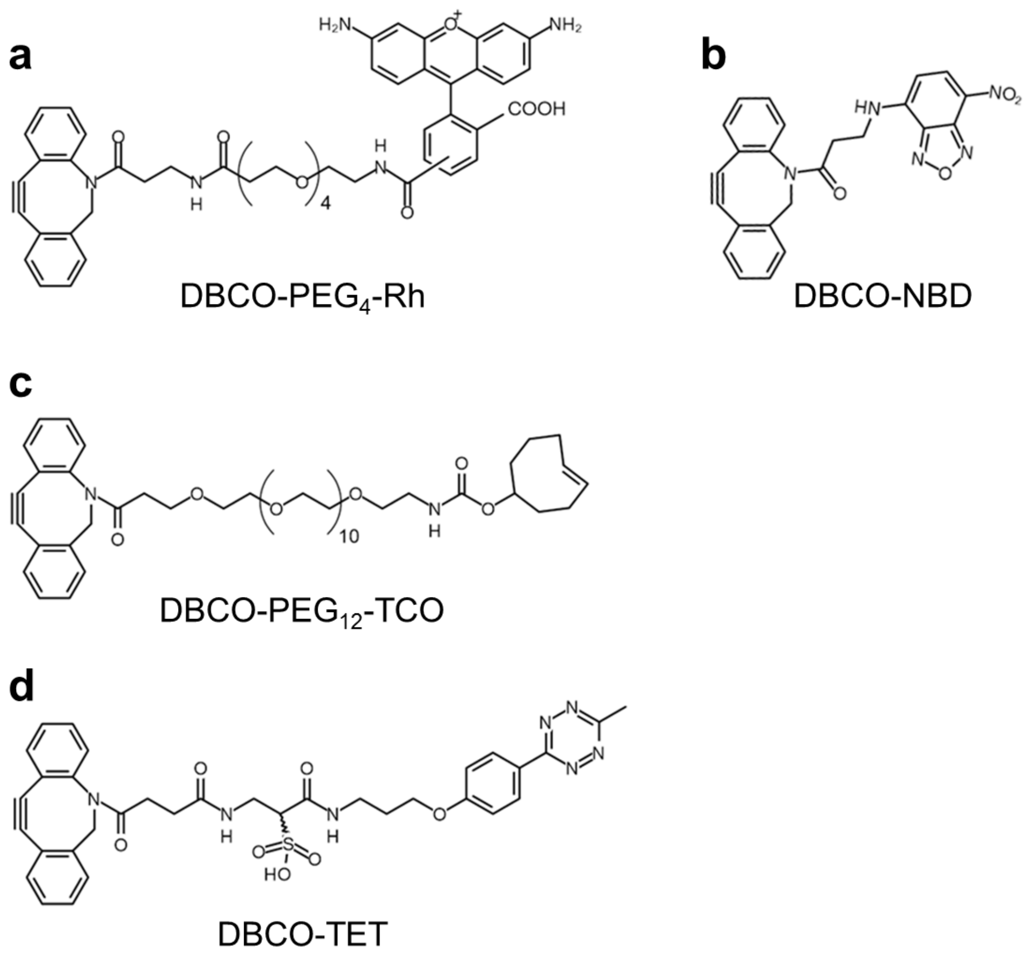

Supplementary Figure 3

Chemical formulas of fluorescent dyes and bifunctional linkers with bio-orthogonal reactivity.

(a) DBCO-PEG$_4$-carboxyrhodamine, a red fluorescent dye that reacts with AZF (b) DBCO-NBD, a green fluorescent dye that reacts with AZF (c) DBCO-PEG$_{12}$-TCO, a bifunctional linker that reacts with AZF and TET (d) DBCO-TET, a bifunctional linker that reacts with AZF and TCO.

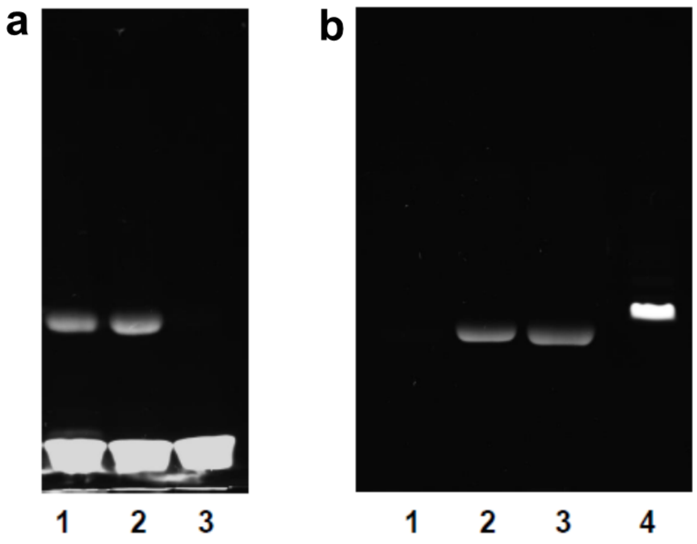

Supplementary Figure 4

Bioorthogonal dye labeling of recoverin variants and purification thereof.

(a) In-gel fluorescence of the dye-labeling reaction mixtures before PD-10 column chromatography. mRec-F23AZF (lane 1), mRec-F158AZF (lane 2), mRec-WT (lane 3) were reacted with DBCO-NBD at RT for 4 hrs, and then analyzed by SDS-PAGE before PD-10 column chromatography. The gel was illuminated at λ_{ex} = 480 nm, and the light above 510 nm was captured. The highly fluorescent lower bands represent unreacted dyes. (b) In-gel fluorescence of dye-labeled recoverin variants after PD-10 purification. No residual dye was observed with mRec-WT (lane 1), mRec-F23NBD (lane 2), or mRec-F158NBD (lane 3). The fluorescent protein mCherry (27 kDa) was run as a reference to recoverin (24 kDa) (lane 4).

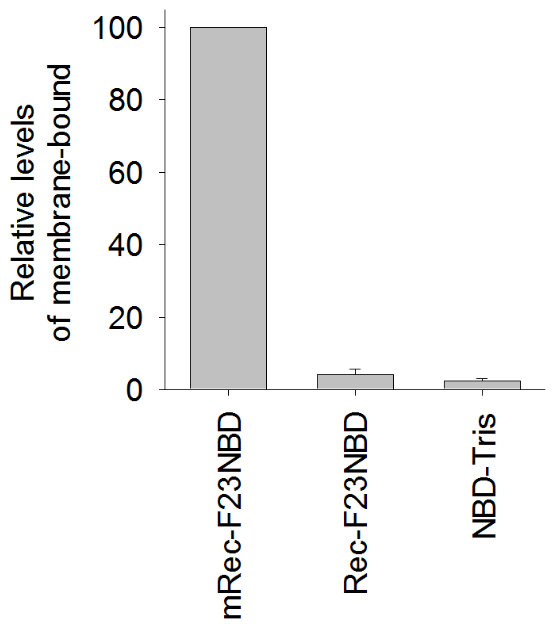

Supplementary Figure 5

Association of NBD-labeled recoverins and free NBD dye with membranes.

Relative mean fluorescence intensity recorded by TIRF microscopy for the binding of NBD-labeled recoverins and NBD-Tris to supported membranes composed with PC:PE (7:3) in the presence of 1 mM Ca^{2+}. To control for free NBD dye binding, nonfluorescent NBD-Cl was reacted with Tris buffer to yield the fluorescent NBD-amino derivative, NBD-Tris.

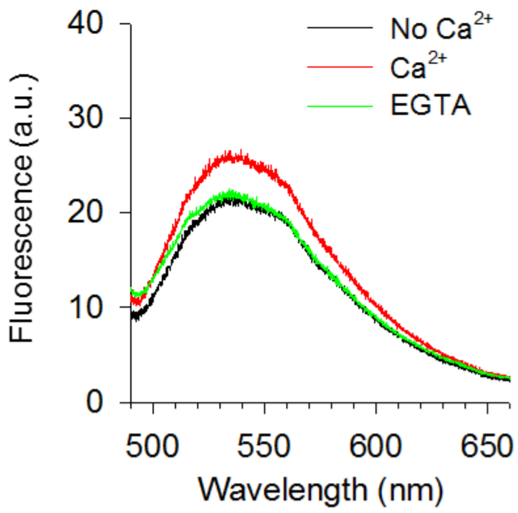

Supplementary Figure 6

Association of mRec-F158NBD with membranes.

Recoverin was labeled site-specifically with NBD for spectrometric measurement of membrane association. Fluorescence emission spectra of 0.1 μM mRec-F158NBD were recorded in the presence of LUVs (0.1 mM total lipids) composed of PC:PE (7:3) in the absence (black) and presence (red) of 1 mM Ca^{2+}. 2 mM EGTA was added after a 60 min incubation of the recoverin-LUV sample with 1 mM Ca^{2+} (green).

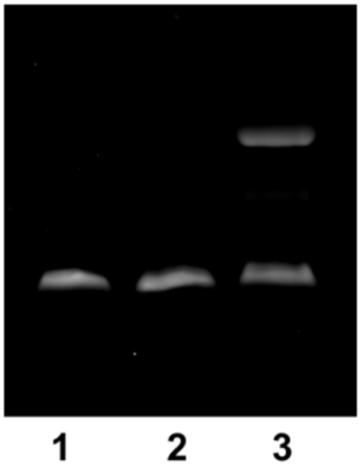

Supplementary Figure 7

Site-specific conjugation of mCherry to recoverin visualized by fluorescent imaging of a SDS polyacrylamide gel.

Lane 1, mCherry-V2AZF; Lane 2, an equimolar mix of mCherry-V2AZF and mRec-F158AZF; Lane 3, reaction mixture of mCherry-V2TCO and mRec-F158TET. The gel was illuminated by visible light to excite the fluorophore of mCherry and imaged with a red emission filter. The molecules run at 27 kDa for mCherry and 52 kDa for the mCherry-mRec conjugate.

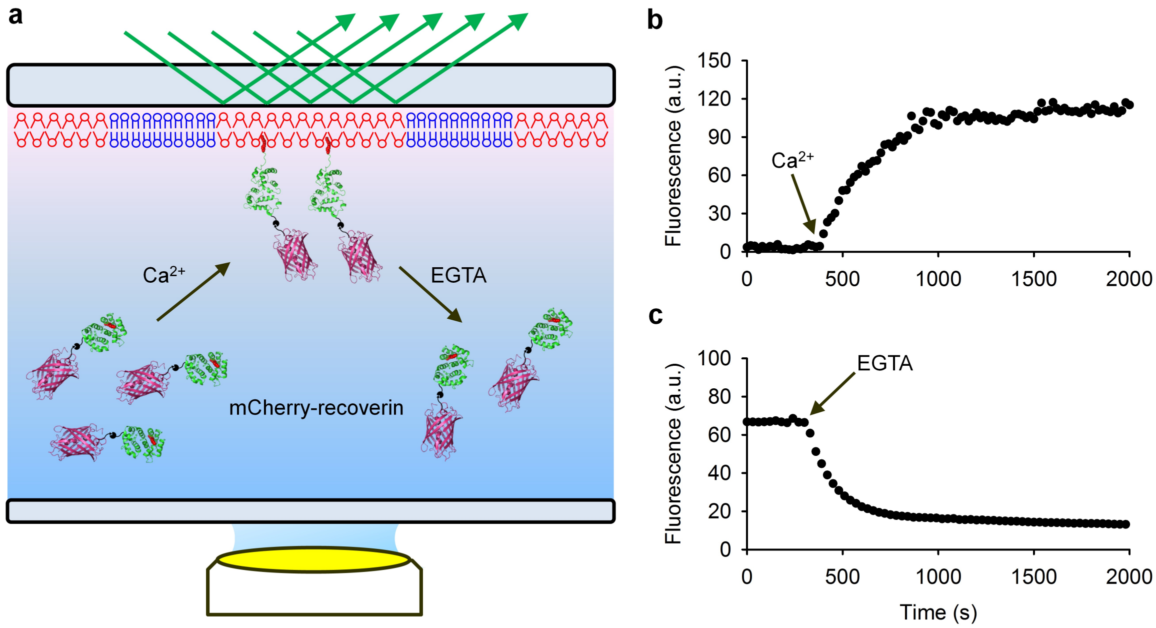

Supplementary Figure 8

Ca^{2+}-responsive reversible translocation of the mCherry-recoverin conjugate to supported membranes.

(a) Schematic of TIRF microscopy approach used to monitor the Ca^{2+}-responsive reversible translocation of the mCherry-recoverin conjugate to a supported membrane. (b) Association of 0.1 μM mCherry-recoverin conjugate to a supported membrane composed of DPPC:DOPC:Cholesterol (2:2:1). The time course of the mean fluorescence intensity as an indicator of recoverin binding to the membrane was recorded after addition of 1 mM Ca^{2+}. (c) Dissociation of the mCherry-recoverin conjugate from the supported membranes after addition of 2 mM EGTA.

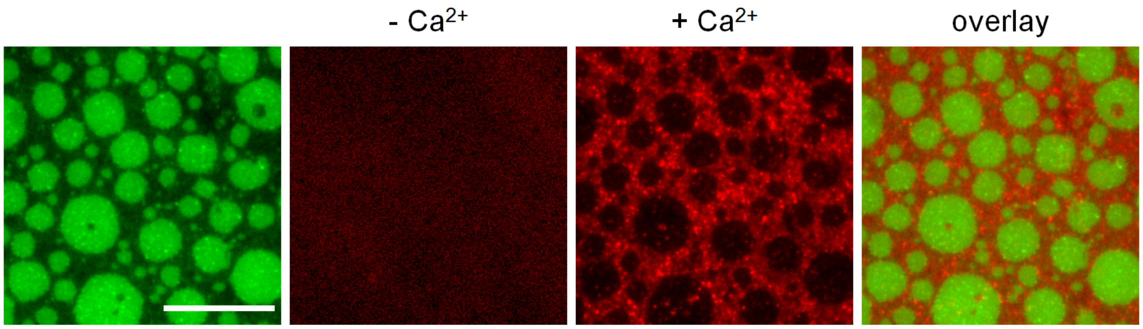

Supplementary Figure 9

Ca^{2+}-dependent binding of the mCherry-recoverin conjugate to supported membranes.

The supported lipid bilayer (left) was composed of DPPC:DOPC:Cholesterol (2:2:1) with coexisting Lo and Ld phases. The membrane was labeled with 0.5 mol% NBD-DPPE which preferentially partitions into Lo phase domains. The conjugate was added to the membrane in the absence (center left) and presence (center right) of 1 mM Ca^{2+}. The overlay image (right) shows that the conjugate associated with membrane regions in the Ld phase in the presence of 1 mM Ca^{2+}. Scale bar is 20 μm.

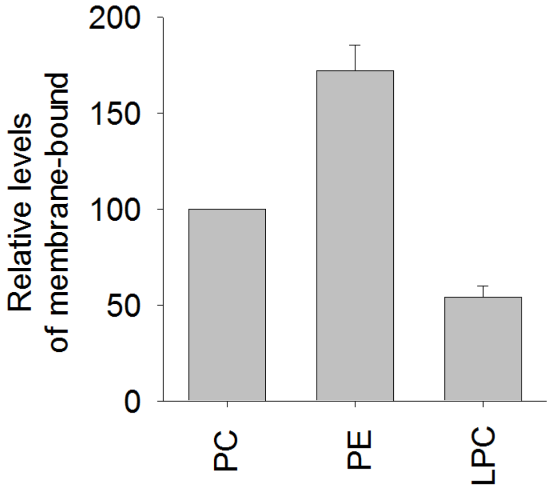

Supplementary Figure 10

Effect of spontaneous membrane curvature on membrane association of the mCherry-recoverin conjugate to supported membranes.

Relative mean fluorescence intensity recorded by TIRF microscopy for the binding of mCherry-recoverin conjugate to supported membranes composed of PC, PC:PE (7:3), or PC:LPC (7:3) in the presence of 1 mM Ca^{2+}.

Supplementary Table 1: Secondary structure contents of mRec and variants without or with Ca^{2+}.

	α-helix	β-sheet	β-turn	unordered
mRec	0.44 / 0.53*	0.15 / 0.08	0.15 / 0.15	0.26 / 0.24
mRec-F23AZF	0.45 / 0.50	0.15 / 0.10	0.16 / 0.16	0.23 / 0.25
mRec-F158AZF	0.43 / 0.54	0.16 / 0.08	0.15 / 0.14	0.27 / 0.24

*Values (fractions of 1) in the absence / presence of 1 mM Ca^{2+} were obtained by deconvolution of the background-corrected CD spectra of each protein using the Dichroweb online CD analysis server.

Supplementary Table 2: Primer sequences used for the recoverin mutagenesis.

Primer	Sequence
NMTtoQE80 F	TTCACACAGAATTCATTAAAGAGGAGAAATTAACTATGTCAGAAGAGGATAAAGCGAAA
NMTtoQE80 R	GTGTGACCGTGTGCTTCTCAAATGCGCTTGGATTCTCACCAATAAAAAACGC
80toRSF F	CCCTTATGCGACTCCTGCATTAGGCTCGAGAAATCATAAAAAATTTATTTGCTTTGTGAG
80toRSF R	GTGTGACCGTGTGCTTCTCAAATGCGCTTGGATTCTCACCAATAAAAAACGC
RecToQE80GFP F	CAATTTCACACAGAATTCATTAAAGAGGAGAAATTAACTATGGGGAACAGCAAGAGTGG
RecToQE80GFP R	CTCAGCTAATCAGTGATGGTGATGGTGATGGAGTTTCTTTTCCTTCAGTTTCTCCTTC
Rec F23amb F	CTGAACACCAAGTAGACGGAGGAGGAG
Rec F23amb R	CTCCTCCTCCGTCTACTTGGTGTTCAG
RecF158amb F	GAAGATCTGGGGATAGTTTGGCAAGAAG
RecF158amb R	CTTCTTGCCAAACTATCCCCAGATCTTC
Fusion RF F	TTCACACAGAATTCATTAAAGAGGAGAAATTAACTATGCGTTCATCACATCATCACC
Fusion RF R	CAGGAGTCCAAGCTCAGCTAATTAAGCTCACATCAGACCAAAGAATTGTTG
mCherryV2amb F	CACTCCTCGGGCTAGTCAAAAGGCGAAG
mCherryV2amb R	CTTCGCCTTTTGACTAGCCCGAGGAGTG
